# The magnetic beads-based sandwich-shaped immune complexes for rapid and sensitive amperometric detection of SOX2 protein

**DOI:** 10.55730/1300-0527.3712

**Published:** 2024-12-16

**Authors:** Göksu ÖZÇELİKAY AKYILDIZ, Mehmet Altay ÜNAL, Seçil GÜLDEN, Sibel A. ÖZKAN

**Affiliations:** 1 Department of Analytical Chemistry, Faculty of Pharmacy, Ankara University, Ankara, Turkiye; 2 Stem Cell Institute, Ankara University, Ankara, Turkiye; 3 Department of Molecular Biology and Genetics, Faculty of Science, Bilkent University, Ankara, Turkiye

**Keywords:** Amperometry, magnetic beads, immunosensor, sandwich-shaped immune complexes, SOX2 protein

## Abstract

Sex-Determining Region Y-box 2 (SOX2) is a transcription factor protein. SOX2 expression is related to lymph node metastasis and distant metastasis in colorectal carcinomas. SOX2 was determined with the first magnetic disposable immunoplatform. The designed sandwich-shaped immune complexes were formed by a capture antibody, SOX2 protein, and biotinylated secondary antibodies (dAb/HRP). The sandwich-shaped immune complex was linked to carboxylic acid functionalized magnetic beads (HOOC-MBs). This magnetic bioconjugate was dropped on the surface of the screen-printed carbon electrode (SPCE). The amperometric measurement was performed at –0.20 V in the presence of hydroquinone (HQ) and H_2_O_2_ against a silver pseudo-reference electrode. The optimization parameters affecting the immunoassay response were evaluated.

The analytical evaluation of the magnetic disposable immunoplatform for the amperometric detection of SOX2 standards was done. The developed immunosensor shows high sensitivity (LOD of 1.37 ng mL^−1^) and a short analysis time (15 min). Potential interfering compounds found in serum samples were tested. The storage stability of magnetic disposable immunoplatform was evaluated. The developed immunosensor was compared with the ELISA method.

## 1. Introduction

Cell type-specific transcription factors control a stem cell’s self-renewal or differentiation [1]. Many studies have focused on these factors, from cancer pathogenesis to treatment [2]. It belongs to the SRY-related HMG-box (SOX) family and is a crucial transcription factor for maintaining differentiation and pluripotent stem cells during embryonic development[3]. Cancer stem cells (CSC) possess advanced tumorigenicity, unlimited self-renewal capacity, and resistance to chemotherapy[4]. Studies conducted in various cancer models before clinical trials have demonstrated that cancer cells expressing SOX2 exhibit distinct characteristics associated with cancer stem cells[5].

Recently, SOX2 expression has been reported to be associated with lymph node metastasis and distant metastasis in colorectal carcinomas and is a poor prognostic factor. The SOX2 gene belongs to the SOXB1 group and is located on chromosome 3q26.3 – q27 [6]. SOX2 protein is a 34.3 kDa transcription factor consisting of 317 amino acids[7]. SOX2 has three main domains: amino terminus, carboxyl terminus, and HMG. One of the most critical functions of SOX2 is to regulate OCT4 expression[8].

In the last two decades, ~20 genes from the SOX gene family have been identified and classified based on their protein specificities [9]. These genes fall into three main families: SOX-B1 (including SOX1, SOX2, and SOX3), SOX-B2 (including SOX14 and SOX21), and SOX-C (including SOX4, SOX11, and SOX12). The discovery and characterization of the SOX2 protein in humans occurred in 1994[6].

SOX2 is a transcription factor that plays a role in preserving the pluripotency of embryonic stem cells and various developmental processes, including lung branching morphogenesis[10]. Analyses of known genes involved in progression and differentiation in tumor gene expression studies have revealed that SOX2 is overexpressed in poorly differentiated cancer subtypes [11]. SOX2 is amplified and overexpressed in various malignancies such as lung, prostate, breast, colon, glioblastoma, ovarian, cervical, and pancreatic cancers[12].

A cancer diagnosis is realized by different techniques such as imaging of tumors, biopsy, enzyme-linked immunosorbent assays (ELISA), mass spectrometry, high-performance liquid chromatography, and so on. Moreover, biosensors are generally an attractive alternative technique because of their affordable, sensitive, and selective features[13].

Electrochemical immunosensors have garnered significant attention due to their sensitivity, simplicity, and cost-effective methods[14]. The electrochemical examination of antigen-antibody interactions enables a rapid, sensitive, selective, and affordable analysis of cancer biomarkers[15]. Compared to the long-adopted ELISA methodology, electrochemical immunosensors have proven advantageous mainly in terms of simplicity, response time, affordability, and in-field application. Electrochemical immunosensors can be divided into unlabelled immunosensors (label-free immunosensors) and labelled immunosensors (sandwich-type immunosensors). Compared with sandwich-type immunosensors, label-free immunosensors have lower sensitivity and selectivity. In addition, the analytical performance of sandwich immunoassays has been enhanced. As the first MBs-based amperometric immunosensor for the sensitive analysis of SOX2, electrochemical biosensors coupled with magnetic microbeads (MBs) were described using a simple approach. To perform a sandwich-type assay, a specific SOX2 capture antibody (CAb) was covalently immobilised on carboxylated magnetic microbeads (COOH-MBs). After lyophilised human SOX2 recombinant protein was captured by the CAb-MBs immunoconjugates, a human SOX2 detector antibody (DAb) recognised SOX2, resulting in a sandwich immunoconjugate that was tagged with a streptavidin-horseradish peroxidase (Strep-HRP) conjugate.

The different immunosensor of SOX2 protein was developed by the scientist [16–19]. Moreover, the different biosensing methods are also developed. Jie Y. et. al. was developed the aggregation-induced electrochemiluminescence (AIECL) emitter for detection of biomarkers. This study shows that AIECL with low cost provided alternative candidates for expensive biomolecules and electrochemiluminescence emitters, opening promising avenues to develop novel ECL systems for biomarkers assays[20,21].

In the present work, the magnetic disposable immunoplatform for determining SOX2 protein was developed. The sandwich-shaped immune complexes involve the capture of Ab, SOX2 protein, and the detection of Ab-HRP. The carboxylated magnetic beads were used as a solid support. The SPCE was used as an electrochemical transducer. The calibration range was linear between 0.01 and 50 ng mL^−1^ with an LOD of 30 pg mL^−1^. The developed immunosensor was also compared with the reference method, ELISA. LOD was found as 200 pg mL^−1^ using ELISA.

## 2. Materials and Methods

### 2.1. Apparatus and electrodes

CHI 812 B instruments (Austin, USA) and Dropsens (Oviedo, Spain) were used for amperometric measurement. The screen-printed carbon electrode (SPCE, DRP-110) and connector cable (DRP-CAC) were supplied from the Dropsens. A magnetic holder (Dynal Mag-2 was bought from Invitrogen-Thermo Fisher (Waltham, MA, USA). A neodymium magnet (AIMAN GZ) embedded homemade holder was used as a platform for the amperometric measurement. The homemade holder fixed the MB-modified SPCE.

A magnetic holding block, Dynal Mag-2 (product No. 12321D, Invitrogen-Thermo Fisher), was employed in magnetic separation steps for the rinsing processes. A pH meter (Basic 20+, Crison) was used to measure pH. The thermo-shaker (MT-100, Universal Labortechnik) was used for incubation. A vortex (Velp Scientifica) was also used to mix the solution. All measurement and preparation procedures were performed at room temperature.

### 2.2. Reagents and solutions

All chemicals have analytical-grade properties. The 0.01 M di-sodium hydrogen phosphate (Na_2_HPO_4_), 0.0027 M potassium chloride (KCl), 0.01 M sodium di-hydrogen phosphate (NaH_2_PO4), 0.137 M sodium chloride (NaCl), 0.0018 M potassium dihydrogen phosphate (KH_2_PO_4_) were used for the preparation of phosphate-buffered saline (0.1 M PBS, pH 7.4). These are supplied by Scharlab (Barcelona, Spain). The buffers were prepared with purified water.

The 2-(N-morpholino)ethanesulfonic acid (MES) and tris(hydroxymethyl)aminomethane (Tris)−HCl were purchased by Scharlab (Barcelona, Spain). The N-hydroxysulfosuccinimide (sulfo-NHS), N-(3-dimethylamino- propyl)-N’-ethylcarbodiimide (EDC), ethanolamine, hydroquinone (HQ), hydrogen peroxide (H_2_O_2_) (30 %, w/v), human serum albumin (HSA), hemoglobin (Hb), and immunoglobulin G (IgG) were supplied from Sigma-Aldrich (St. Louis, Missouri, USA). The commercial blocking solution (BB) (a ready-to-use, PBS solution of 1% w/v purified casein) was supplied from Thermo Fisher Scientific (Waltham, Massachusetts, USA).

The carboxylated magnetic microbeads (COOH-MBs, 2.7 μm Ø, 10 mg mL^−1^, Dynabeads M − 270, Cat. No: 14305D) were purchased from Invitrogen-Thermo Fisher Scientific (Waltham, Massachusetts, USA). The SimpleStep ELISA® Kit (product No ab245707) consisting of human SOX2 capture antibody (CAb), human SOX2 detector antibody (DAb), human SOX2 lyophilized recombinant protein (SOX2), and streptavidin peroxidase conjugate (Strep-HRP) were supplied by Abcam (Cambridge, UK).

### 2.3. Preparation of the MBs-based immunoplatform

The COOH-MBs suspension (3 μL) added to the microcentrifuge (1.5 mL) was placed in a thermo shaker (25 °C, 950 rpm) and washed twice with 50 μL of 0.025 M MES buffer (pH 5.0). To remove the supernatant after all relevant steps, the COOH-MBs were placed in a magnetic holder for 3 min to remove the supernatant part from the magnetic beads.

Then, COOH-MBs were incubated with freshly prepared sulfo-NHS/EDC mixture solution for 35 min in a thermo shaker (950 rpm, 25 °C), and then the previous washing process was applied. 1/25 CAb solution prepared in MES was incubated for 15 min after being washed twice with MES. 1 M ethanolamine solution prepared in phosphate buffer (pH 8.0) was incubated for 60 min in the thermo-shaker (25 °C, 950 rpm) for blocking of unreacted group. Then, the as-modified MBs were washed with TRIS buffer and twice with PBS (pH 7.4). The CAb/COOH–MBs were kept in the refrigerator by being suspended in PBS.

### 2.4. Immunoassay procedure

The CAb-COOH-MBs were applied in a single 15-min step by incubating the mixture solution consisting of 1/25 dAb/HRP and SOX2 standard (prepared in BB: PBS (2:1)) for 15 min in the thermo-shaker (25 °C, 950 rpm), before washing twice with BB solution. Finally, the magnetic microcarrier was resuspended in 50 μL of 0.05 M sodium phosphate buffer solution (pH 6.0), and amperometric detection was performed.

### 2.5. Amperometric measurements

CAb-COOH-MBs were dropped onto the SPCE surface fixed on the homemade holder. The CAb-COOH-MBs/SPCE was immersed in the 0.05 M sodium phosphate buffer (pH 6.0) in the presence of 0.1 mM hydroquinone (HQ)[22]. For the amperometric measurement, the detection potential (–0.2 V) was applied in the stirring conditions in the presence of the Ag pseudo-reference electrode. The 50 μL of 0.1 M H_2_O_2_ (30 %, w/v) was added to the buffer solution when the current reached steady. Therefore, the current resulting from the enzymatic reduction of H_2_O_2_ mediated by HQ was recorded [23].

### 2.6. The expression and purification of His-tagged SOX2 Protein

Expression of recombinant SOX2 protein is done by plasmid vectors that encode the SOX2 protein. These plasmids are then introduced into the host bacteria, and the SOX2 protein expression is induced in the living bacteria. For this purpose, E. coli bacterium containing plasmid vectors pQE and pREP4 was used to express the N-terminal 6xHis-tagged SOX2 protein. A single colony was acquired from a previously prepared bacterial glycerol stock and inoculated into a 3 mL LB broth, followed by the addition of 3 μL Kanamycin and 3 μL Carbenicillin. This colony was incubated overnight using a shaker at 225 rpm at 37 °C.

Overnight-grown culture was transferred to 250 mL LB broth containing 250 μL Kanamycin and 250 μL Carbenicillin. After that, the culture was incubated inside a shaker at 225 rpm at 37 °C until it reached the optical density of 0.6 at 600 nm. To induce the SOX2 expression, 250 μL of 1 mM IPTG was added, and the culture was further incubated for 4 h at 37 °C using a shaker at the same speed as before. After a 5-min incubation on ice, the culture was centrifuged at 3500 rpm for 20 min at 4 °C. The pellet obtained after the centrifugation was stored at –80 °C for later use.

The bacterial pellet stored at –80 °C was resuspended for the purification part by adding a 1 mL Lysis Buffer A and gently pipetting the mixture. The bacterial cells expressing the target protein are lysed by adding the buffer. The solution was centrifuged at 10.000 rpm for 20 min at +4 °C. The supernatant was collected, and lysate was obtained. A metal ion affinity chromatography method called Nickel-affinity chromatography was used for the purification of the His-tagged protein from the lysate. For this aim, the lysate was mixed with Ni^+2^-NTA(Nitrilotriacetic acid) agarose resin in a falcon using a shaker for 1 h. A setup containing column chromatography and a peristaltic pump (Ismatec™ MS-4/6 Reglo Digital Pump) was utilized for the rest of the procedure.

To equilibrate the setup, lysis buffer A (0.06 Tris base, 28.66 g Gu-HCl, 0.69 g NaH_2_PO_4_.H_2_O (pH:8.0)) was flowed through the column using the peristaltic pump to ensure a consistent flow rate which is one drop per 3 seconds, then discarded. Afterwards, the lysate mixed with resin was poured inside the column, and lysis buffer B (0.12 g Tris base, 48.0 g urea, 0.69 g NaH_2_PO_4_.H_2_O(pH = 8.0)) was run through the setup in the same way. For the washing step, wash buffer C (0.12 g Tris base, 48.0 g urea, 1.38 g NaH_2_PO_4_.H_2_O (pH = 6.3)) was used. As a final step of the protein purification, elution buffer D (0.12 g Tris base, 48.0 g urea, 1.38 g NaH_2_PO_4_.H_2_O (pH=6.3)) and elution buffer E (0.12 g Tris base, 48.0 g urea, 1.38 g NaH_2_PO_4_.H_2_O (pH=4.5)) was passed through the column consecutively thereby allowing the purified protein to be collected in 2 mL tubes. Finally, an Invitrogen Qubit Fluorometer was used to measure the concentration of the purified protein [24].

## 3. Results and discussion

**Figure 1:**
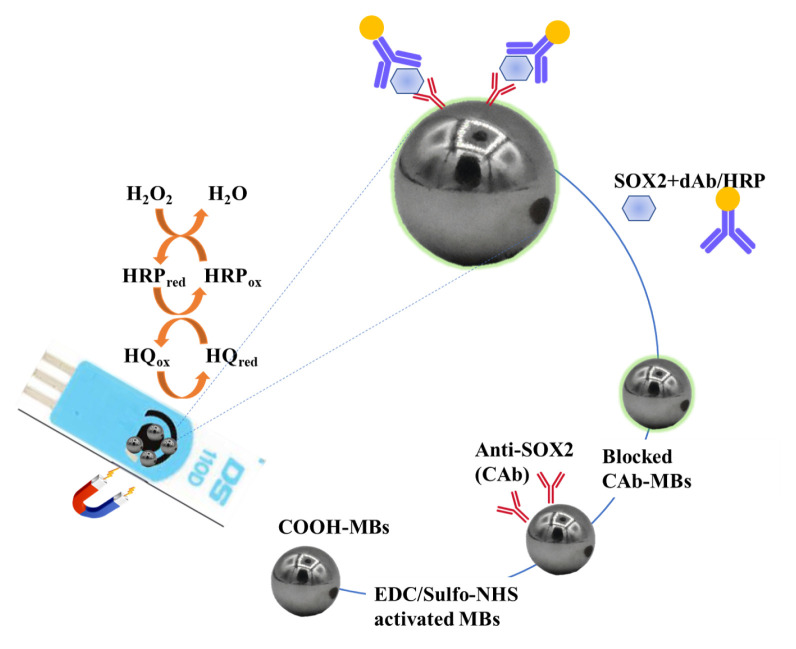
The preparation scheme of the developed magnetic disposable immunoplatform.

The scheme of the developed immunoplatform is shown in Figure 1. The immunoassay involved the sandwich-shaped immune complexes consisting of CAb, SOX2 protein, and biotinylated detection antibodies (dAb/HRP). The COOH-MBs were activated by carbodiimide chemistry, and then the SOX2-specific CAb was covalently immobilized to activate the COOH-MBs. The CAb/COOH-MBs were exposed to the 1 M ethanolamine to block free-active sites on the surface. Finally, the SOX2 and dAb/HRP were immobilized to magneto-immunoconjugates onto SPCEs.

### 3.1. Optimization of variables

**Figure 2:**
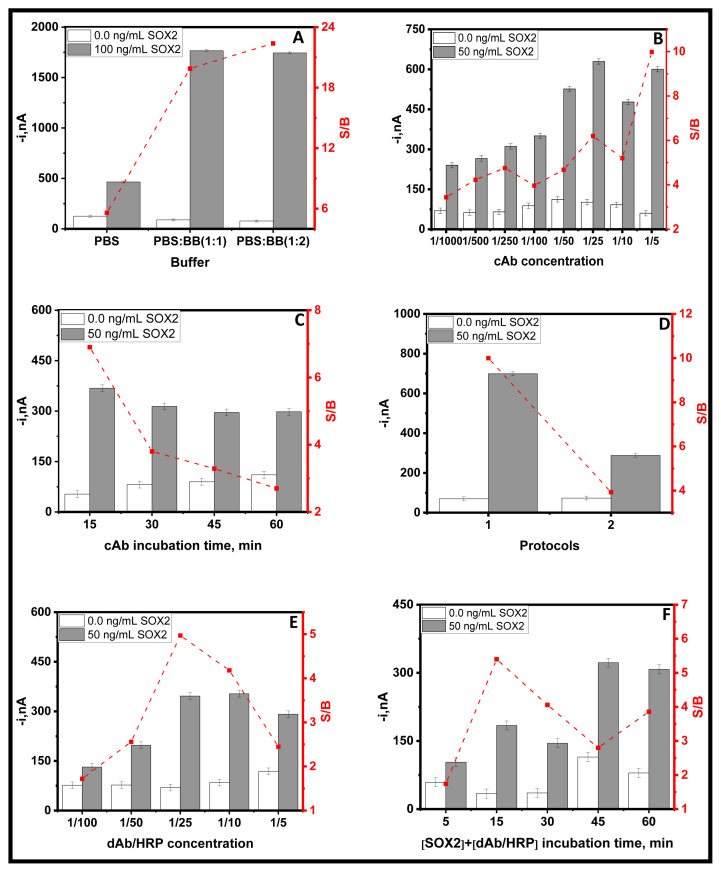
The optimization parameters of the sandwich-shaped immune complexes (A) the buffer solution used to prepare the standard, (B) the CAb concentration used to modify the MBs, (C) the incubation time of the CAb, (D) protocols tested to perform the sandwich-shaped immune complexes, (E) dAb/HRP concentration, (F) incubation time of the SOX2+dAb/HRP.

The impact of important parameters on the immunosensor response has been assessed in Table 1 and Figure 2. These variables included (a) buffer, (b) CAb concentration, (c) incubation time of CAb, (d) protocols, (e) dAb/HRP concentration, and (f) incubation time of mixture solution of SOX2 + dAb/HRP. The selection of the optimal parameter was based on the ratio of amperometric responses measured in the presence (S, grey bars) and absence (B, white bars) of a SOX2 standard solution, aiming for a higher S/B ratio (in red line).

As seen in Figure 2A, the buffer solution plays a determinant role in the S/B ratio, and the best results are achieved when the SOX2 standard is prepared in a mixture solution of blocking buffer(BB) (2) and PBS (1). The effect of CAb concentration on the immunosensor response was tested by incubating the CAb solutions with concentrations ranging from 1/1000 to 1/5 for 30 min (25 °C, 950 rpm) to activated COOH-MBs. Figure 2B shows that the highest S/B ratio was found using 1/5 CAb concentration. However, as a compromise between sensitivity and cost per test, a concentration of 1/250 CAb was selected. The incubation time of 1/250 CAb solution was tested between 15 and 60 min (25 °C, 950 rpm). As can be seen in Figure 2C, a 15 min incubation was sufficient to obtain the highest S/B ratio. The high B signals obtained when 1/250 CAb are immobilized (Figure 2B) or short incubation times are used for its immobilization (Figure 2C) could be due to nonspecific adsorptions of dAb/HRP on MBs or on CAb. The number of incubation steps was optimized to form MBs-based sandwich-shaped immune complexes, as shown in Table 2, to decide the optimum protocol.

All the assayed protocols consisted of incubating the CAb-MBs immunoconjugates in solutions containing the immunoreagents for 30 min. Results in Figure 2D show that the larger S/B ratio was obtained using the single-step protocol (protocol 1) when all reagents were mixed with SOX2 standard and 1/10 dAb/HRP. This protocol has significant advantages due to its simplicity and short analysis time. Furthermore, the concentration of dAb/HRP was evaluated by testing dilutions ranging from 1/100 to 1/5 (Figure 2E). The S/B ratio raised with the dAb/HRP concentration up to 1/25 and dropped for larger concentrations regardless of the specific current increase due to the relatively larger increase of the nonspecific current. Thus, the highest S/B ratio was obtained for 1/25. It was found that the optimal incubation time for the only incubation step involved was 15 min (Figure 2F).

### 3.2. Analytical evaluation of the MBs-based sandwich-shaped immune complexes

**Figure 3:**
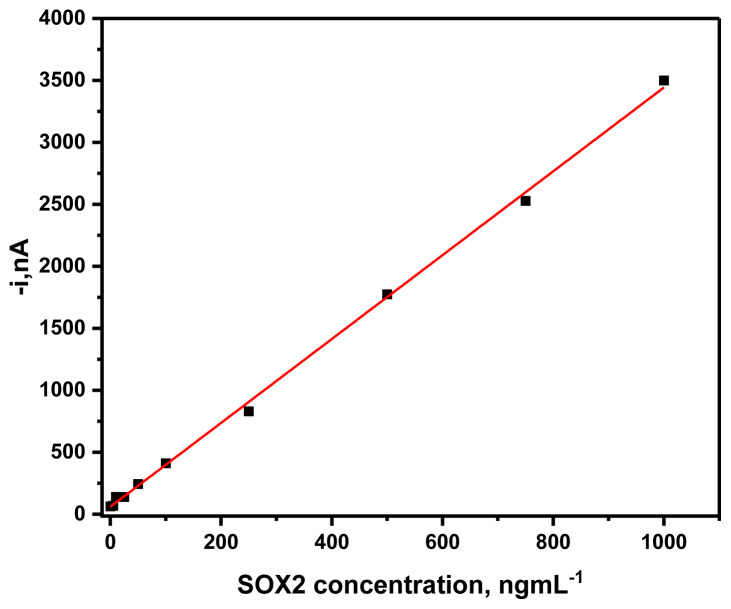
Using the MBs-based sandwich-shaped immune complexes, a calibration plot was constructed for the amperometric response and the SOX2 standard concentration.

The calibration graph was obtained by plotting the variation in the measured amperometric response vs. the recombinant SOX2 standard concentration using the MBs-based sandwich-shaped immune complexes prepared in the optimized conditions (Figure 3). The standard curve was linear between 5.0 and 1000 ng mL^−1^. Moreover, a linear calibration plot (R^2^ = 0.999) was drawn between the amperometric response and the SOX2 standard concentration, obtaining slope (3.38 ± 0.0426) mL nA ng^−1^, and intercept (59.99 ± 18.51) nA values.

The limits of detection (LOD) and quantification (LOQ) were calculated to 3×sd/m and 10 × sd/m, respectively (sd = standard deviation of the amperometric response of SOX2 protein, and m = the slope of the calibration equation). A LOD of 1.37 ng mL^−1^ and LOQ of 4.58 ng mL^−1^ were obtained.

The relative standard deviation (RSD) was evaluated for the repeatability of magnetic platform-assisted immunoplatform using an amperometric response of 250 ng mL^−1^ SOX2 protein. The 10.0% RSD confirmed the acceptable repeatability of the developed magnetic platform-assisted immune platform. The RSD% value was obtained from the six experiments’ mean(m) and standard deviation (sd). The data are presented in Table 3.

The storage stability of the cAb-MBs conjugates was tested. The cAb-MBs were stored in filtered PBS at 4 ^o^C. The platform was prepared before the measurements were taken.

The storage stability of the developed MBs-based sandwich-shaped immune complexes was evaluated for 15 days. There was no significant difference in the amperometric ratio (S/B) value of 0.0 (blank, B) and 5 ng mL^−1^ SOX2 (signal, S) during 15 days.

This shows that the prepared MBs-based sandwich-shaped immune complexes provide time-efficient detection. The data are presented in Table 4.

### 3.3. Selectivity studies of the magnetic platform-assisted immunoplatform

**Figure 4:**
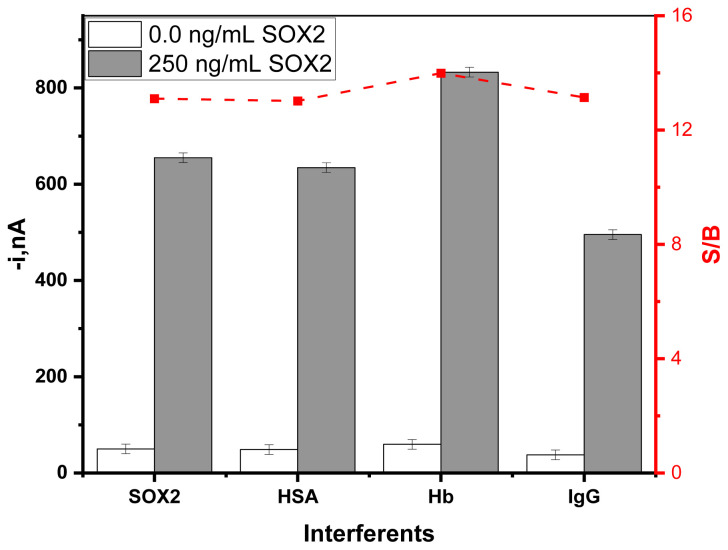
The comparison of the amperometric response of MBs-based sandwich-shaped immune complexes in the presence and absence of interferents for 0 (white bars) and 250 ng mL^−1^(grey bars) SOX2 protein.

The amperometric response of recombinant SOX2 protein in the absence and presence of interfering proteins (5 mg mL^−1^ hemoglobin (Hb), 50 mg mL^−1^ albumin from human serum albumin (HSA), and 1.0 mg mL^−1^ human immunoglobulin G (hIgG)) found in serum were examined in Figure 4.

It was found that there was no significant interference in the amperometric ratio (S/B) of signal (S) and blank(B), thus confirming the excellent selectivity of the pair of antibodies involved in the immunoplatform. However, Hemoglobin (Hb) significantly altered the S/B ratio. Hb, exhibiting peroxidase activity, is a source of some active oxygen species. Its intrinsic peroxidase activity can explain more significant signals obtained when Hb is present in the solution [25].

### 3.4. Determination of purified SOX2 protein

**Figure 5:**
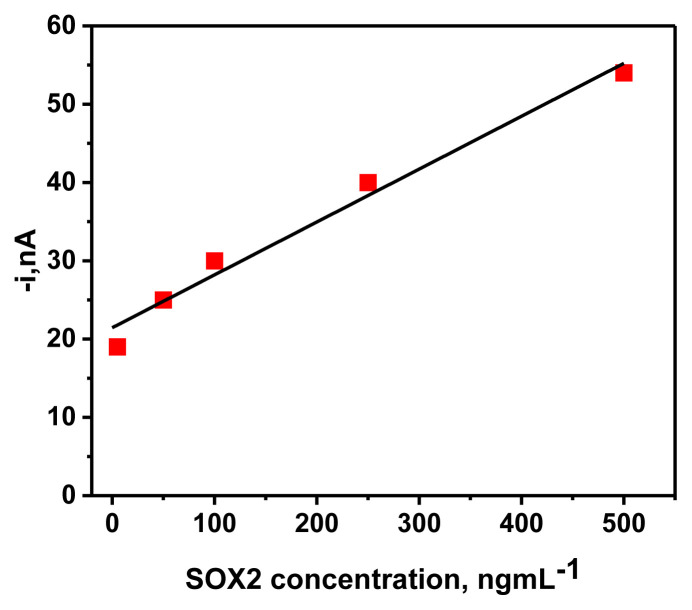
The calibration curve was drawn between amperometric response versus purified SOX2 protein.

The linear calibration curve was obtained by plotting the amperometric response vs. the purified SOX2 protein concentration using the MBs-based sandwich-shaped immune complexes prepared in the optimized conditions. The standard curve was linear between 5.0 and 500 ng mL^−1^. Moreover, a linear calibration curve (R^2^ = 0.980) was drawn according to the relation between the amperometric response versus the purified SOX2 protein concentration, obtaining slope (0.0676 ± 1.32) mL nA ng^−1^ and intercept (21.322 ± 3.8) nA values (Figure 5). A LOD (1.35 ng mL^−1^) and LOQ (4.5 ng mL^−1^) were obtained.

### 3.5. ELISA measurements

**Figure 6:**
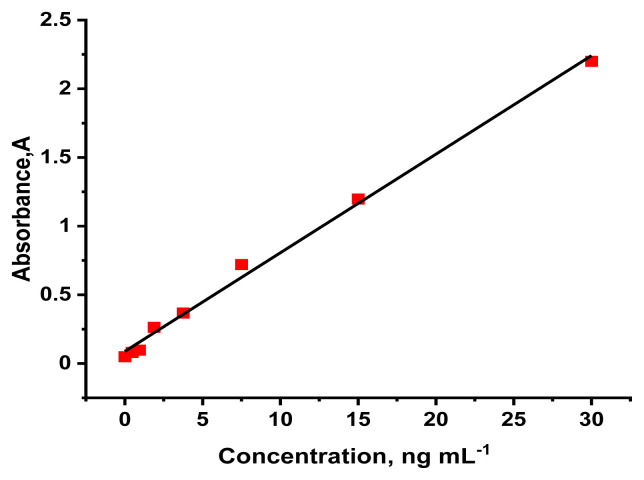
The calibration plot of SOX2 standards with ELISA.

The SOX2 was measured with the reference method (ELISA). The standard curve was drawn between 0.47 and 30 ng mL^−1^ for ELISA methods. A linear calibration plot (R^2^ = 0.995) was drawn between the absorbance and the SOX2 standard concentration, obtaining slope (0.0718 ± 0.0021), and intercept (0.0883 ± 0.0257) values (Figure 6). Accordingly, the obtained LOD (0.128 ng mL^−1^) and LOQ (0.428 ng mL^−1^) values were found.

The absorbances of the purified SOX2 protein were measured. Moreover, the concentration of purified SOX2 protein was found to be 2.79 ng mL^−^1, 5.35 ng mL^−^1, 11.11 ng mL^−^1, and 23.03 ng mL^−1^.

## 4. Conclusions

A novel magnetic platform-assisted immunoplatform has been developed to rapidly and sensitively determine SOX2 to help diagnose diseases. The immunoplatform is based on an enzyme-labelled sandwich-shaped immune complex that employs a CAb, SOX2 protein, and a dAb/HRP to perform amperometric detection on SPCE. The developed immunosensor shows high sensitivity (LOD of 1.37 ng mL^−1^) and a short analysis time. The developed immunosensor was compared to the previously published paper. SOX2 protein was determined with an immunosensor by Ozcan et al. [16]. The silanization agent linked indium tin oxide- polyethylene terephthalate (ITO-PET) was used as the working electrode. The linear range was 0.02 pg mL^−1^ −2 pg mL^−1^ with an LOD of 0.013 pg mL^−1^. The disposable ITO-based electrochemical immunosensor was developed to determine SOX2 protein by Aydın E.B et al. [17]. The carboxyethylsilanetriol was used as an electrode modifier. The linear detection range was found between 25 fg mL^−1^ and 2 pg mL^−1^, with a 7 fg mL^−1^ LOD. Tarimeri N. et al. developed ITO-based biosensor. ITO-PET electrode surfaces were modified with 3-glycidoxypropyl trimethoxysilane. The linear range of SOX2 was found as 0.625–62.5 pg mL^−1^(LOD of 0.16 pg mL^−1^) [18].A microfluidic electrochemical immunosensor was fabricated by Regiart M. et al. [19]. The gold electrode was used as a detector. The calibration range was linear between 0.01 and 50 ng mL^−1^ with an LOD of 30 pg mL^−1^. The developed immunosensor was also compared with the reference method, ELISA. LOD was found as 200 pg mL^−1^ using ELISA. Moreover, the magnetic platform-assisted immunoplatform was compared with the ELISA methodology. The results were in harmony with each other, proving that the biosensor could be applied to the human serum, and it is undoubtedly very advantageous for clinical use. ELISA analysis time was 90 min, whereas the magnetic platform-assisted immunoplatform was only 15 min. Moreover, the developed immune platform has a wide determination range (5.0–1000 ng mL^−1^), whereas the ELISA calibration range (0.47–30 ng mL^−1^) was narrow. The electrochemical immunosensor offered several attractive advantages compared to the ELISA, such as high stability, portability, selectivity, reusability, and sensitivity. In conclusion, the immunoplatform represents a more cost-effective approach and suitable point-of-care usage.

Moreover, this device could be used for clinical diagnosis and prognosis of several kinds of carcinomas in human serum samples.

## Figures and Tables

**Table 1:** The evaluation of the optimization parameters using the MBs-based sandwich-shaped immune complexes.

Parameters	Checked range	Optimal value
Buffer (a)	PBS, BB	A mixture of BB (2) and PBS (1)
CAb concentration (b)	1/1000 – 1/5	1/250
Incubation time of CAb, min (c)	15–60	15
Protocols (d)	1–2	1
dAb/HRP concentration (e)	1/100–1/5	1/25
Incubation time of mixture solution of SOX2 + dAb/HRP, min (f)	5–60	15

**Table 2:** The optimization of protocols to develop an immunological platform.

Protocol	Steps (all using incubation steps of 30 min)
**1**	a single step-a mixture solution consisting of SOX2 standard and 1/10 dAb/HRP
**2**	Two steps-(1) the SOX2 standard solution and then (2) 1/10 dAb/HRP

**Table 3:** The data of the repeatability of the developed magnetic platform-assisted immune platform.

	S (-i,nA)
**1.repeat**	853.8
**2.repeat**	714.7
**3.repeat**	743.0
**4.repeat**	830.5
**5.repeat**	903.6
**6.repeat**	916.4

**m**	**827**
**sd**	**82.77**
**%RSD**	**10.00**

**Table 4:** The data of storage stability of the developed MBs-based sandwich-shaped immune complexes according to days.

Days	S(-i,nA)	B(-i,nA)	S/B
**1. Day**	45	4	11
**7. Day**	50	4	12.5
**15. Day**	65	5	13
